# Measurement of Technetium-99m Sestamibi Signals in Rats Administered a Mitochondrial Uncoupler and in a Rat Model of Heart Failure

**DOI:** 10.1371/journal.pone.0117091

**Published:** 2015-01-16

**Authors:** Akira Kawamoto, Takao Kato, Tetsuo Shioi, Junji Okuda, Tsuneaki Kawashima, Yodo Tamaki, Shinichiro Niizuma, Yohei Tanada, Genzou Takemura, Michiko Narazaki, Tetsuya Matsuda, Takeshi Kimura

**Affiliations:** 1 Department of Cardiovascular Medicine, Graduate School of Medicine, Kyoto University, Kyoto, Japan; 2 Cardiovascular Center, Kitano Hospital, The Tazuke Kofukai Medical Research Institute, Osaka, Japan; 3 Division of Cardiology, Gifu University, Graduate School of Medicine, Gifu, Japan; 4 Department of Systems Science, Graduate School of Informatics, Kyoto University, Kyoto, Japan; Rutgers New Jersey Medical School, UNITED STATES

## Abstract

**Background:**

Many methods have been used to assess mitochondrial function. Technetium-99m sestamibi (^99m^Tc-MIBI), a lipophilic cation, is rapidly incorporated into myocardial cells by diffusion and mainly localizes to the mitochondria. The purpose of this study was to investigate whether measurement of ^99m^Tc-MIBI signals in animal models could be used as a tool to quantify mitochondrial membrane potential at the organ level.

**Methods and Results:**

We analyzed ^99m^Tc-MIBI signals in Sprague-Dawley (SD) rat hearts perfused with carbonyl cyanide *m*-chlorophenylhydrazone (CCCP), a mitochondrial uncoupler known to reduce the mitochondrial membrane potential. ^99m^Tc-MIBI signals could be used to detect changes in the mitochondrial membrane potential with sensitivity comparable to that obtained by two-photon laser microscopy with the cationic probe tetramethylrhodamine ethyl ester (TMRE). We also measured ^99m^Tc-MIBI signals in the hearts of SD rats administered CCCP (4 mg/kg intraperitoneally) or vehicle. ^99m^Tc-MIBI signals decreased in rat hearts administered CCCP, and the ATP content, as measured by ^31^P magnetic resonance spectroscopy, decreased simultaneously. Next, we administered ^99m^Tc-MIBI to Dahl salt-sensitive rats fed a high-salt diet, which leads to hypertension and heart failure. The ^99m^Tc-MIBI signal per heart tissue weight was inversely correlated with heart weight, cardiac function, and the expression of atrial natriuretic factor, a marker of heart failure, and positively correlated with the accumulation of labeled fatty acid analog. The ^99m^Tc-MIBI signal per liver tissue weight was lower than that per heart tissue weight.

**Conclusion:**

Measurement of ^99m^Tc-MIBI signals can be an effective tool for semiquantitative investigation of cardiac mitochondrial membrane potential in the SD rat model by using a chemical to decrease the mitochondrial membrane potential. The ^99m^Tc-MIBI signal per heart tissue weight was inversely correlated with the severity of heart failure in the Dahl rat model.

## Introduction

Technetium-99m sestamibi (^99m^Tc-MIBI) is a lipophilic cation [1]. The cellular uptake of this lipophilic cation is governed by the Nernstian equation across the plasma and mitochondrial membranes in cells [2, 3]. Because cells have a negative transmembrane potential and a highly negative mitochondrial membrane potential, ^99m^Tc-MIBI accumulates in the mitochondria in living cells [2] [3]. In neonatal cultured cardiomyocytes, most ^99m^Tc-MIBI was concentrated in mitochondria in a dose-dependent manner [4]. In subcellular fractions, the ^99m^Tc-MIBI content correlates with the expression of the mitochondrial inner matrix enzyme malate dehydrogenase and with mitochondrial substrates in isolated perfused hearts [5]. In addition, more than 90% of ^99m^Tc-MIBI is localized to the mitochondrial fraction and is in proportion with the activity of succinate dehydrogenase, a mitochondrial marker, in hearts excised from animals 10 min after intravenous bolus administration [6]. Therefore, the amount of retained probe is also proportional to the volume of mitochondrial matrix [3] and the amounts of mitochondrial inner membrane lipids if the probe binds to these lipids.

Mitochondria play key roles in ATP synthesis via the Krebs cycle and oxidative phosphorylation, maintenance of redox homeostasis, regulation of intracellular calcium, and cell-death related processes and apoptosis [7]. Abnormalities in any of these processes can be defined as or linked to mitochondrial dysfunction. Many methods are used to assess mitochondrial function and dysfunction using isolated mitochondria, intact cells, or *in situ* techniques, each of which has advantages and disadvantages related to experimental control and physiological relevance [8]. Methods for the assessment of isolated mitochondria are generally well established and enable quantitative and qualitative assessment of mitochondrial function, without interference from cytosolic factors. However, these methods lack a cellular context; they often require large amounts of samples as well as appropriate substrates and experimental conditions for specific purposes. Methods involving intact cells allow the study of mitochondria in an undisturbed cellular environment with preserved localization; however, many reagents and substrates are impermeable and unable to modify mitochondria directly, and mitochondria are not directly accessible to the full range of extracellular substrates, hormones, and cell-cell interactions. Although *in situ* techniques preserve the full complexity of the system, that complexity makes it difficult to distinguish between mitochondrial dysfunction and other confounding factors [8].

The mitochondrial membrane potential is governed by the balance of inward and outward ion fluxes in the inner mitochondrial membrane [9]. In isolated mitochondria, membrane potential is calculated by monitoring the distribution of the cations between the incubation medium and mitochondrial matrix, and these are generally reported to be high, *i.e*., 172 mV [10], 196 mV [11], and 210–220 mV in isolated liver mitochondria [12] and around 190 mV in isolated brain mitochondria [13]). Impedance spectroscopy with parallel pH monitoring showed a potential of 144 mV in isolated heart mitochondria [14]. For intact cells, the fluorescent membrane-permeant cationic probe tetramethylrhodamine ethyl ester (TMRE) is a probe that is frequently used probes for analyzing mitochondrial membrane potential [8]. Methods using intact cells are as accurate as the methods using isolated mitochondria [12, 15, 16]. Intact cells show lower mitochondrial membrane potential in the range of 81 to 140 mV [9], *i.e*., 81 mV in cultured neuroblastoma [17], 117 mV for embryonic heart cells [4], and 100–140 mV in perfused hearts depending on workload and substrates [18]. These differences in measured potential may be, in part, due to differences in experimental conditions and mitochondrial function among species and tissues. Moreover, these results were not obtained from working organs.

The purpose of this study was to test whether ^99m^Tc-MIBI signals in animal models could be used as a tool to quantify mitochondrial membrane potential at the organ level. To this end, we compared the signals of TMRE and ^99m^Tc-MIBI in *ex vivo* perfused hearts treated with carbonyl cyanide *m*-chlorophenylhydrazone (CCCP) to decrease the mitochondrial membrane potential. We also measured ^99m^Tc-MIBI signals in the hearts of rats administered CCCP or vehicle. Furthermore, considering the numerous studies examining the role of mitochondrial dysfunction in heart failure [7, 19–23], we measured ^99m^Tc-MIBI signals in a rat model of cardiac hypertrophy and failure.

## Methods

### 1. Animals and materials

Eight-week-old male Sprague-Dawley (SD) rats (weight: 280–290 g, Shimizu Laboratory Supplies, Kyoto, Japan) were used for *ex vivo* perfused heart experiments and administration of CCCP in an animal model. As a model of cardiac hypertrophy and heart failure, we used Dahl salt-sensitive (DS) rats. Inbred male DS rats (Japan SLC, Hamamatsu, Shizuoka, Japan) were fed a low-salt (LS) diet (0.3% NaCl) until the age of 6 weeks, when they were switched to a high-salt (HS) diet (8% NaCl) [24]. As we reported previously [21], DS rats that were fed an HS diet developed hypertension and left ventricular hypertrophy (LVH) at 11 weeks of age and subsequent heart failure around 18 weeks of age. DS rats fed only the LS diet, which did not lead to development of hypertension or LVH, were used as controls. Animal care and experiments were approved by the Institutional Animal Care and Use Committee of Kyoto University and conducted according to the Guide for the Care and Use of Laboratory Animals published by the United States National Institutes of Health.

CCCP (Wako Pure Chemical Industries, Osaka, Japan) was diluted in 100% dimethyl sulfoxide (DMSO) for a 10 mM stock solution. ^99m^Tc-MIBI and ^125^I-15-*(p*-iodophenyl)-9-*R,S*-methylpentadecanoic acid (^125^I-9MPA), a fatty acid tracer, were purchased from FUJIFILM RI Pharma Co., Ltd. (Tokyo, Japan).

### 2. Measurement of mitochondrial membrane potentials in perfused hearts using TMRE

After heart excision, the ascending aortas were cannulated with a customized needle, and hearts were perfused using the Langendorff technique with oxygenated (95% O_2_) Tyrode’s solution containing 134 mM Na, 4 mM KCl, 1.2 mM MgSO_4_, 1.2 mM NaH_2_PO_4_, 10 mM HEPES, 11 mM D-glucose, and 2 mM CaCl_2_ (pH = 7.4 adjusted with 1 M NaOH: warmed to 37°C). After an initial perfusion for stabilization, the buffer was switched to Tyrode’s solution containing 100 nM TMRE for 10 min, followed by a 10-min perfusion with dye-free solution. Next, the hearts were placed in circular glass-bottom dishes and perfused further with a solution containing 2,3-butanedione monoxime (BDM) for 5 min to eliminate contraction-induced movement for two-photon imaging [25, 26]. The images were acquired after the heartbeat was arrested. After stabilization for 20 min, the hearts were perfused with a solution containing CCCP (1 or 0.1 μM) or vehicle for 20 min (n = 3–4 per group). Images were recorded with a Zeiss LSM510 laser scanning microscope (Carl Zeiss MicroImaging GmbH, Jena, Germany). Illumination for two-photon excitation was provided by a mode-locked Ti sapphire laser. The excitation wavelength was 810 nm. Hearts were imaged through a Zeiss 40 × (1.30 numerical aperture) oil-immersion objective lens with a working distance of 180–200 μm. Emitted light collection was collected through two photomultiplier tubes fitted with band-pass filters for 565–615 nm light. Regions of interest were drawn over a part of an individual cell, and signals within the regions were collected over time. Image analysis was performed using Image-J (http://imagej.nih.gov/ij/). The TMRE brightness was quantified using converted black-and-white images.

### 3. Measurement of ^99m^Tc-MIBI signals in perfused hearts

Hearts were excised and perfused as described above. After a stabilization period of 10 min, hearts were perfused with Tyrode’s solution at a dose of 12 MBq (324.3 μCi) ^99m^Tc-MIBI for 5 min (uptake). Hearts were then perfused with Tyrode’s solution without the tracer for 35 min (clearance). At 20 min after starting ^99m^Tc-MIBI perfusion, CCCP (1 or 0.1 μM) or vehicle was perfused for 20 min (n = 3–4 per group). Radiotracer activity was measured at 10-min intervals throughout the experiment by a lead-collimated sodium iodide scintillation detector placed 3 cm from the heart.

### 4. Measurement of ^99m^Tc-MIBI signals in excised hearts in CCCP-administered rats

CCCP was previously reported to decrease hepatic ATP production when administered to rats at a dose of 4 mg/kg with no mortality and at 5 mg/kg with 11% mortality [27]. Rats were randomly divided into three groups. One group was euthanized 15 min after a dose of 12.5 MBq (337.8 μCi) ^99m^Tc-MIBI injection (n = 6). The other two groups were administered 4 mg/kg CCCP (CCCP group; n = 7) or vehicle (vehicle group; n = 7) by intraperitoneal (i.p.) injection 90 min after the same dose of ^99m^Tc-MIBI injection and were euthanized after an additional 90 min (180 min after the ^99m^Tc-MIBI injection). Hearts were excised and weighed, and radioactivity was measured between 110 and 170 keV with an auto-well gamma counter (Cobra2^TM^ Auto-gamma, Packard). ^99m^Tc-MIBI signals were corrected for physical decay (half-life = 6 h).

### 5. Effect of CCCP on myocardial phosphocreatine and ATP levels

To investigate the effect of CCCP on energy production in mitochondria, phosphocreatine (PCr) and ATP levels were measured using *in situ* ^31^P magnetic resonance (MR) spectroscopy at 30 min after i.p. injection of 4 mg/kg CCCP or vehicle (CCCP: n = 5, vehicle: n = 4). Experiments were performed as previously reported [21]. Briefly, the animals were anesthetized with an i.p. injection of pentobarbital (30 mg/kg). Gradient echo transverse hydrogen-1 (^1^H) MR images were obtained to define regions of interest. ^31^P MR spectroscopy of the anterior left ventricular wall was conducted using a surface coil (20-mm diameter) and depth-resolved surface coil spectroscopy [28]. Phosphorus metabolism was calculated by computer integration of the areas under the respective peaks (Scion Image 4.0, Scion Corporation, Frederick, MD, USA).

### 6. ^99m^Tc-MIBI signals in a rat model of cardiac hypertrophy

Sixty-two DS rats fed an HS diet and 20 DS rats fed an LS diet were used in this experiment. By 18 weeks of age, 10 rats in the HS group died of heart failure; therefore, 20 DS rats fed LS diet and the 52 remaining DS rats fed HS diet were fasted overnight and injected with 12.5 MBq (337.8 μCi) of ^99m^Tc-MIBI and 0.37 MBq (10 μCi) of ^125^I-9MPA, which reflects β-oxidation activity in mitochondria [29–31], via a tail vein. Ten DS rats fed an LS diet and 26 DS rats fed an HS diet were euthanized by decapitation 45 min after the injection. Another 10 DS rats fed an LS diet and 26 DS rats fed an HS diet were euthanized 6 h after the injection. The hearts and livers were removed and weighed, blood samples were collected, and radioisotopic activity was measured using a scintillation counter. The radioisotopic activity of ^99m^Tc-MIBI was measured just after euthanasia. The radioisotopic activity of ^125^I-9MPA (half-life = 59.4 days) was measured 10 days after the injection, as described [21]. All analyses were performed after correction for the physical decay of radioisotopes.

### 7. Echocardiography

A transthoracic echocardiographic analysis was performed as previously reported [21, 24].

### 8. Quantitative reverse transcription (RT)-polymerase chain reaction (PCR) for measurement of atrial natriuretic factor (ANF)

Total RNA was isolated from the heart tissue by acid guanidinium thiocyanate-phenol-chloroform extraction. Quantitative RT-PCR was carried out as described previously [21] using the primers listed in Table 1. *ANF* mRNA levels were standardized using the 18S ribosomal RNA as an internal control.

**Table 1:** Primer sequences used in real time quantitative RT-PCR.

**Gene**	**Forward**	**Reverse**	**GenBank Entry**
18S ribosomal RNA	AGTCCCTGCCCTTTGTACACA	CGATCCGAGGGCCTCACTA	M11188
Atrial natriuretic factor	CCGATAGATTCTGCCCTCTTGAA	CCCGAAGCAGCTTGATCTTC	M27498.1

### 9. Transmission electron microscopy

Cardiac tissue from the left ventricle (n = 3 in each group) was quickly cut into 1mm-cubes, immersion-fixed with 2.5% glutaraldehyde in 0.1 M phosphate buffer (pH 7.4) overnight at 4°C, and fixed in 1% buffered osmium tetroxide. Specimens were dehydrated through a graded ethanol series and embedded in epoxy resin. Ultrathin slices (90 nm) were double-stained with uranyl acetate and lead citrate and observed under an electron microscope (H-800, Hitachi, Tokyo, Japan). Morphometrical analyses were performed as previously described [32, 33]. In brief, uniform sampling of 10 electron micrographs was utilized for the morphometric evaluation of each group. Five random fields, micrographed at 20,000 × magnification from of each five blocks, were printed at a final magnification of 50,000 × and analyzed on composite grids as described previously [33] to calculate the density of mitochondria (number per 100 μm^2^) and the size (μm^2^) of mitochondria within cardiomyocytes.

### 10. Statistical analysis

All data are expressed as the mean ± standard error of the mean (SEM). Differences between the groups were analyzed by ANOVA followed by post hoc comparisons with the Bonferroni test. When there were two crossed factors, differences between groups were analyzed by two-way factorial ANOVA followed by post hoc comparisons with the Bonferroni test. Spearman’s rank correlation tests were conducted to assess the relationships between nonparametric variables. In all tests, *P* values of less than 0.05 were considered significant.

## Results

### 1. Effects of CCCP on ^99m^Tc-MIBI signals in *ex vivo* perfused heart

**Figure 1:**
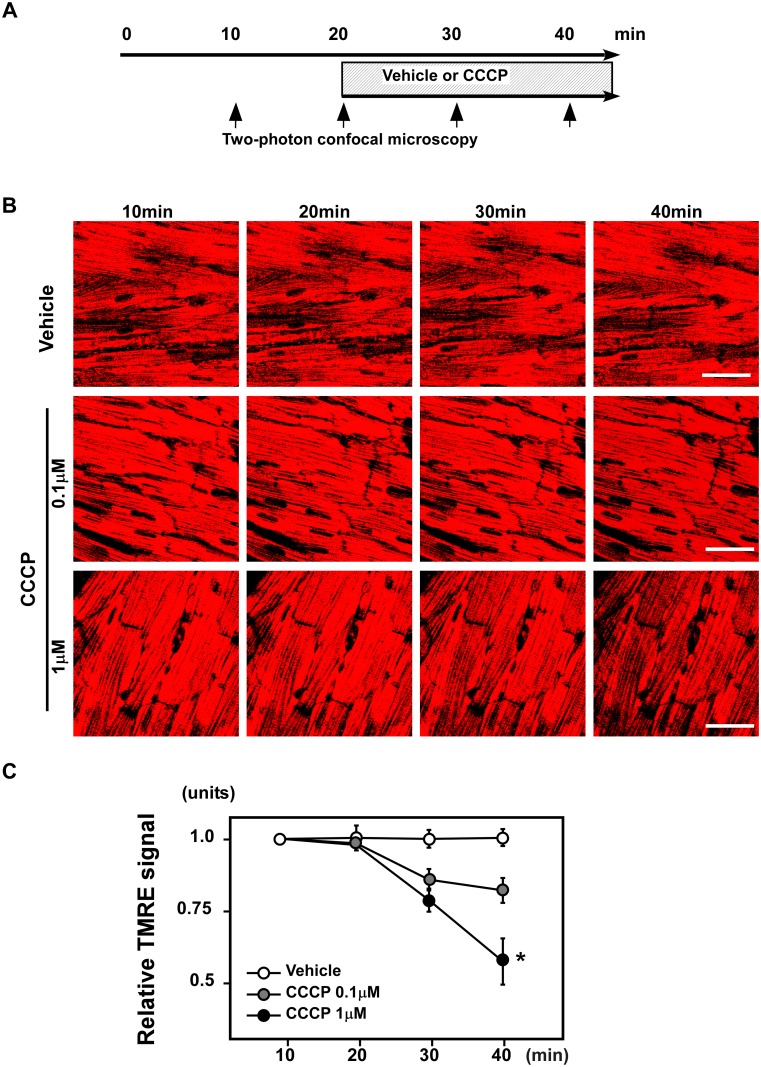
CCCP decreased TMRE signals in *ex vivo* perfused hearts. (A) Schematic of the experiment. Hearts were perfused with TMRE, and the signals were observed using two-photon laser microscopy. The images were obtained serially from hearts perfused with buffer containing vehicle or CCCP (0.1 or 1 μM). (B) Representative images of TMRE signals. Scale bar: 50μm. (C) CCCP decreased the TMRE signals of perfused hearts in a dose-dependent manner 20 min after CCCP perfusion (n = 3–4 per group). *P < 0.05 versus vehicle.

**1–1. CCCP decreased the TMRE signals in ex vivo perfused hearts.** To assess mitochondrial membrane potentials, we first employed TMRE, a well-characterized cationic probe. Hearts were perfused with TMRE, and the signal was observed using two-photon laser microscopy (Fig. 1A). Images were obtained serially from hearts perfused with buffer containing vehicle or CCCP (0.1 or 1 μM; Fig. 1B). CCCP decreased the TMRE signals in perfused hearts in a dose-dependent manner 20 min after CCCP perfusion (Fig. 1C).

**Figure 2:**
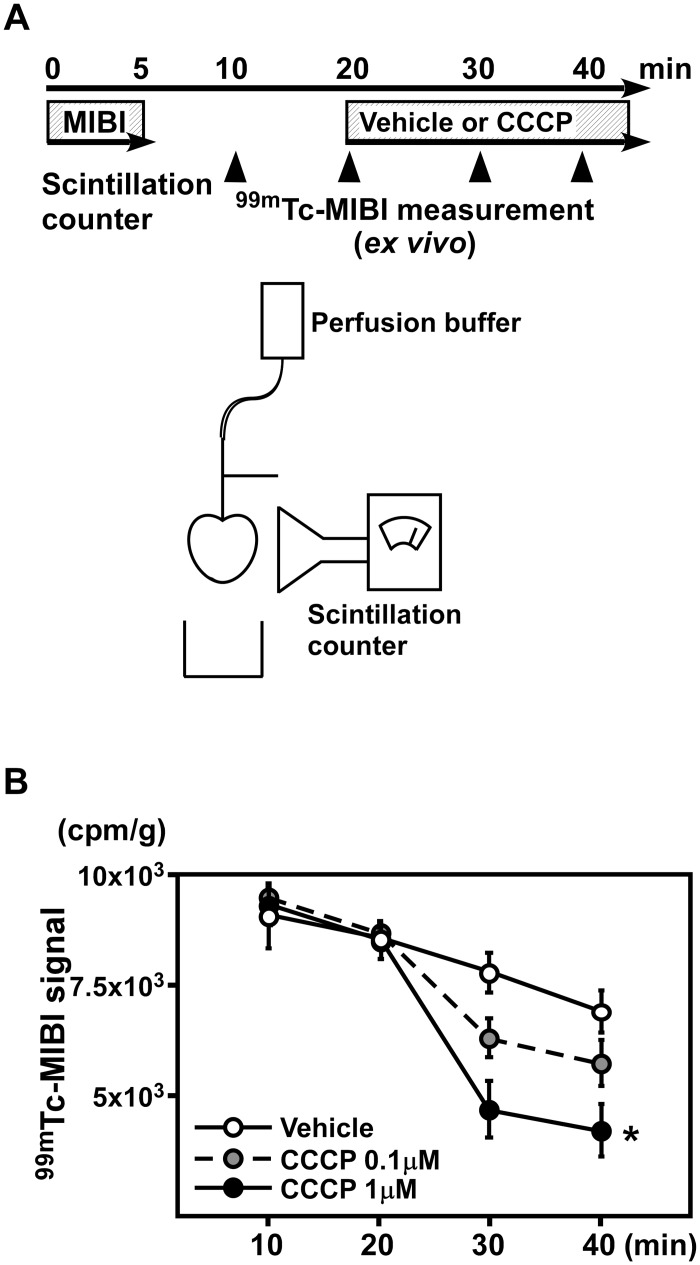
CCP decreased ^99m^Tc-MIBI signals in perfused hearts. (A) A schematic of the experiment. ^99m^Tc-MIBI signals were serially analyzed in perfused hearts. (B) The signals of ^99m^Tc-MIBI were decreased after perfusion of the same doses of CCCP used for the TMRE experiment. (n = 3–4 per group). *P < 0.05 versus vehicle.

**1–2.^99m^Tc-MIBI signals in perfused hearts were decreased by CCCP.** Next, we serially analyzed the ^99m^Tc-MIBI signals in perfused hearts (Fig. 2A). We compared the signals after correction for the physical decay of radioisotopes and found that ^99m^Tc-MIBI signals were decreased after perfusion with the same doses of CCCP used for the TMRE experiment (Fig. 2B). Thus, ^99m^Tc-MIBI was able to detect changes in the mitochondrial membrane potential of the heart tissue with sensitivity similar to that of TMRE.

### 2. Effects of CCCP on ^99m^Tc-MIBI signals in rats

**Figure 3:**
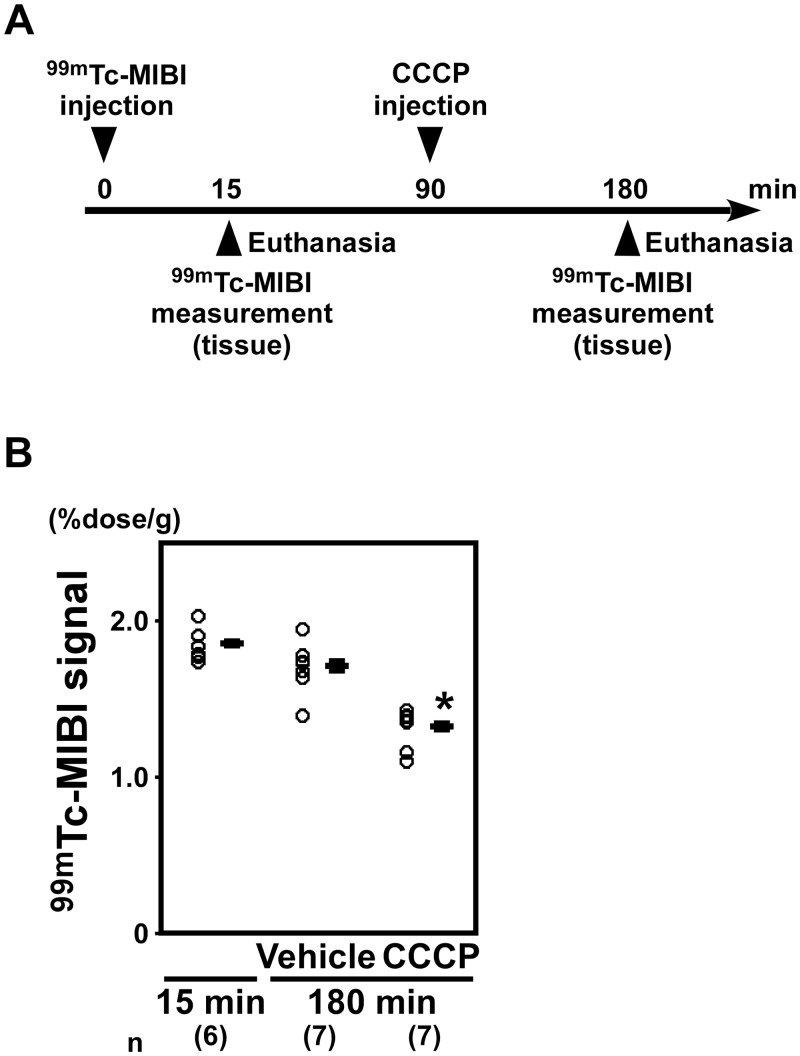
^99m^Tc-MIBI signals were decreased in the hearts of rats administered CCCP. (A) A schematic of the experiment to investigate whether CCCP decreased the ^99m^Tc-MIBI signals in rats. (B) At 180 min after ^99m^Tc-MIBI injection, the ^99m^Tc-MIBI signals of the hearts in the CCCP group were significantly lower than those in the rats administered vehicle (n = 7 in each group). *P < 0.05 versus vehicle.

**2–1. ^99m^Tc-MIBI signals were decreased in the hearts of rats administered CCCP.** To investigate whether CCCP decreased the ^99m^Tc-MIBI signals in rats, we analyzed the radioisotope activity of ****excised heart tissue from rats administered CCCP (Fig. 3A). At 180 min after ^99m^Tc-MIBI injection, the ^99m^Tc-MIBI signals from the hearts in the CCCP group were significantly lower than those in the vehicle group (CCCP: 1.320 ± 0.016%dose/g; vehicle: 1.708 ± 0.029%dose/g; *P* < 0.05; Fig. 3B).

**Figure 4:**
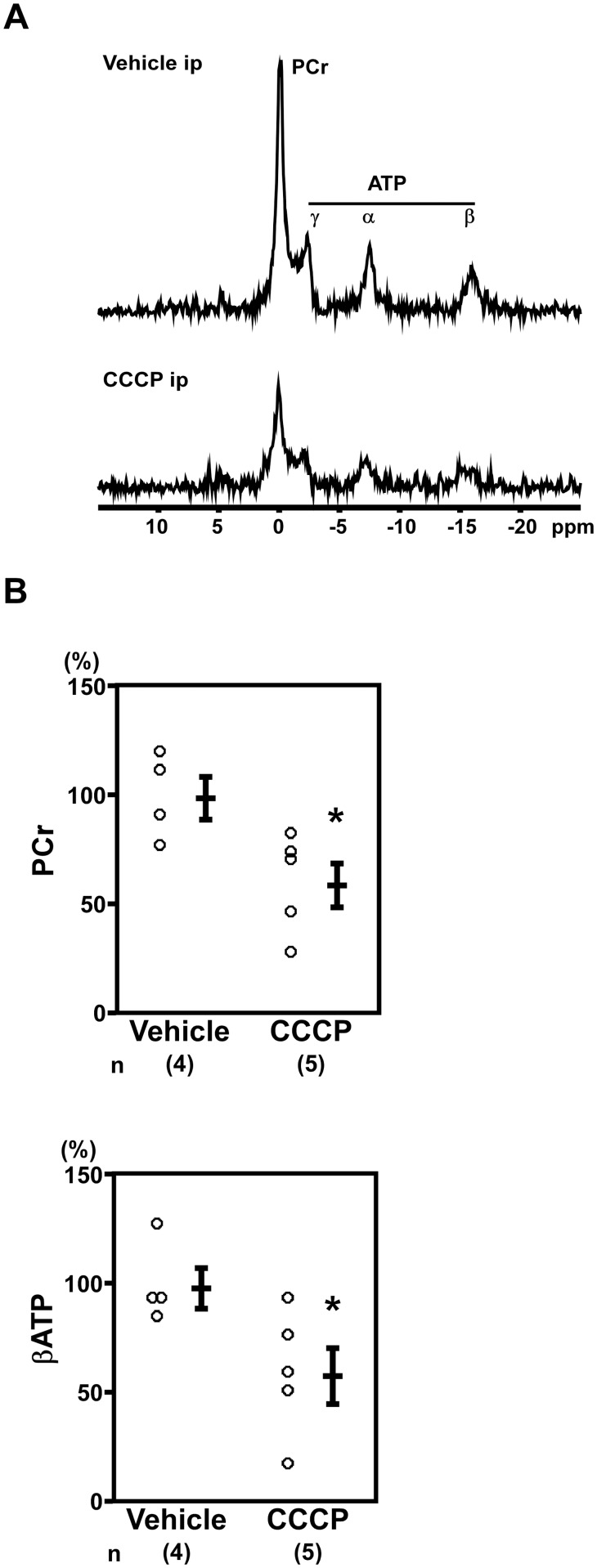
Phosphocreatine and ATP were decreased in the hearts CCCP-administered rats. (A) Representative images of *in situ* cardiac ^31^P magnetic resonance spectra. ppm, parts per million. (B) The rats administered CCCP showed decreased PCr and βATP (Vehicle: n = 4, CCCP: n = 5). *P < 0.05 versus vehicle-administered rats.

**2–2. CCCP decreased *in situ* cardiac phosphocreatine levels.** Next, we examined the effects of CCCP on *in situ* cardiac phosphocreatine and ATP levels to clarify the relationship between ^99m^Tc-MIBI signals and the energy status of the heart. *In situ* cardiac ^31^P MR spectroscopy revealed that CCCP-administered rats had decreased phosphocreatine (vehicle: 100.0% ± 9.7%; CCCP: 60.1% ± 10.1%; n = 4–5, *P* < 0.05; Fig. 4A, 4B) and βATP (vehicle: 100.0% ± 9.4%; CCCP: 59.6% ± 12.9%; n = 4–5, *P* < 0.05; Fig. 4A, 4B) levels.

### 3. Analysis of ^99m^Tc-MIBI signals in a rat model of heart failure

**Figure 5:**
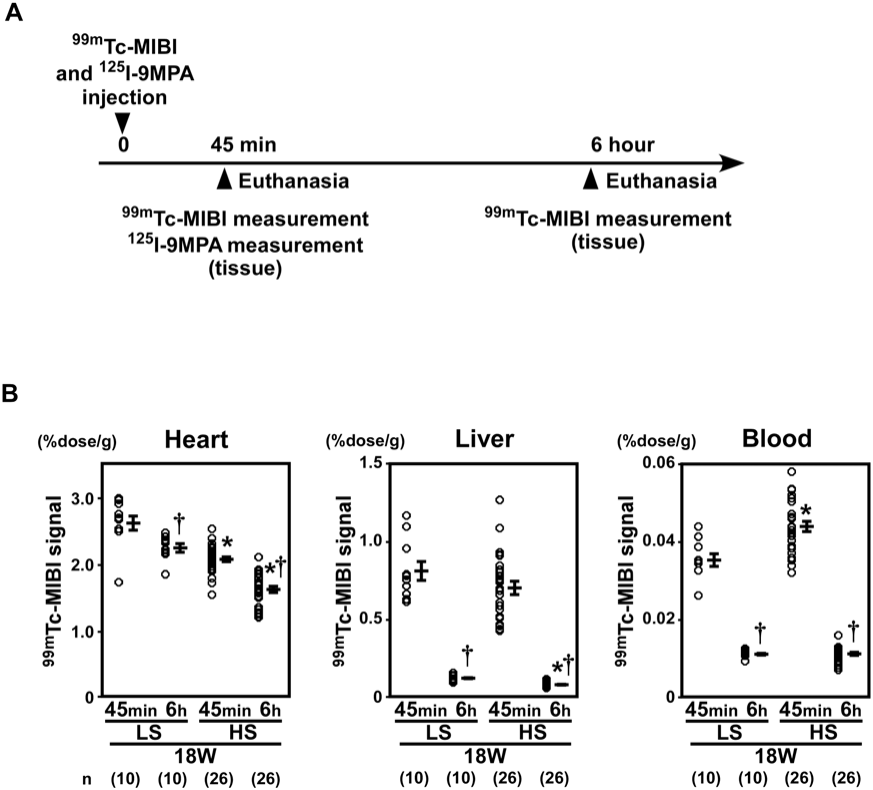
^99m^Tc-MIBI signals were decreased in the hearts of DS rats fed a high-salt diet. (A) A schematic representation of the experiments used to analyze ^99m^Tc-MIBI and ^125^I-9MPA signals in DS rats. Analyses of ^99m^Tc-MIBI and ^125^I-9MPA signals in DS rats were performed at 45 min and 6h after injection. (B) At 45 min after injection, the ^99m^Tc-MIBI signals were lower in DS rats fed an HS diet than those in DS rats fed an LS diet. In the DS rat fed an HS diet and an LS diet, ^99m^Tc-MIBI signal per heart tissue weight decreased 6 h after the injection compared to 45 min after the injection (left panel) after correction for physical decay. In DS rats fed an HS diet with heart failure at 18 weeks of age, ^99m^Tc-MIBI signal per heart tissue weight decreased compared to those in control ****rats fed an LS diet at 6 h after injection. Signals for ^99m^Tc-MIBI in the liver (middle panel) or blood (right panel) were low in both LS and HS group at 6 h after injection. (LS, 45 min: n = 10, 6 h: n = 10; HS, 45 min: n = 26, 6h: n = 26). LS, low-salt; HS, high-salt. *P < 0.05 versus LS. †*P* < 0.05 versus the early phase group.

**3–1. ^99m^Tc-MIBI signals were decreased in the hearts of DS rats fed an HS diet.** DS rats that were fed an HS diet developed hypertension (Table 2) and LVH at 11 weeks of age (Table 3) and subsequent heart failure around 18 weeks of age (Tables 3 and 4). DS rats fed only the LS diet, which did not lead to development of hypertension (Table 2) or LVH (Tables 3 and 4), were used as controls. At 45 min after injection, the ^99m^Tc-MIBI signals in the heart were lower in DS rats fed an HS diet than those in DS rats fed an LS diet (Fig.5A and 5B, left panel). In DS rats fed an HS diet and an LS diet, the ^99m^Tc-MIBI signal per heart tissue weight decreased 6 h after the injection compared to 45 min after the injection (Fig. 5B, left panel) after correction for physical decay. In DS rats fed an HS diet with heart failure at 18 weeks of age, the ^99m^Tc-MIBI signal per heart tissue weight decreased compared to those in control ****rats fed an LS diet at 6 h after injection (Fig. 5B, left panel). Signals for ^99m^Tc-MIBI in the liver or blood were low in both LS and HS group at 6 h after injection (Fig. 5B, middle and right panel).

**Table 2:** Body weight, pulse rate and blood pressure of 11 week-old DS rats.

	**11 weeks old**	
	**LS**	**HS**
Number of animals	20	52
Body weight (g)	367 ± 4	359 ± 2
Pulse rate (bpm)	373 ± 7	413 ± 4*
Systolic blood pressure (mmHg)	125 ± 3	187 ± 2*
Diastolic blood pressure (mmHg)	93 ± 3	139 ± 2*

Values are expressed as the mean ± SEM.

*P < 0.05 versus LS.

*DS rats*, Dahl salt-sensitive rats; *LS*, rats fed a low-salt diet; *HS*, rats fed a high-salt diet;

SEM, standard error of the mean.

**Table 3:** Echocardiographic data of DS rats.

** **	**11 week-old**		**18 week-old**	
** **	**LS**	**HS**	**LS**	**HS**
Number of animals	20	52	20	52
Heart rate (bpm)	336 ± 11	369 ± 8*	333 ± 8	405 ± 10*
Diastolic PWT (mm)	1.46 ± 0.04	1.95 ± 0.04*	1.26 ± 0.05	1.39 ± 0.03*
LVDd (mm)	7.22 ± 0.21	6.56 ± 0.11*	8.25 ± 0.16	7.42 ± 0.11*
LVDs (mm)	3.27 ± 0.27	2.29 ± 0.11*	4.21 ± 0.16	4.49 ± 0.13
FS (%)	55.8 ± 2.8	65.6 ± 1.3*	49.2 ± 1.2	39.5 ± 1.4*

Values are expressed as the mean ± SEM.

* *P* < 0.05 versus LS at the same age.

*DS rats*, Dahl salt-sensitive rats; *LS*, rats fed a low-salt diet; *HS*, rats fed a high-salt diet;

*PWT*, posterior wall thickness; *LVDd*, left ventricular diastolic dimension;

*LVDs,* left ventricular systolic dimension*; FS*, fractional shortening;

*SEM,* standard error of the mean.

**Table 4:** Postmortem analysis of Dahl rats at 18 weeks old.

	**LS**		**HS**	
	**45 min**	**6 h**	**45 min**	**6 h**
Number of animals	10	10	26	26
Body weight (g)	431 ± 8	433 ± 5	377 ± 5*	380 ± 5*
Heart weight (g)	1.12 ± 0.03	1.11 ± 0.02	1.48 ± 0.02*	1.46 ± 0.02*
Heart weight/Body weight (g/kg)	2.60 ± 0.04	2.57 ± 0.03	3.95 ± 0.08*	3.85 ± 0.08*
Liver weight (g)	10.72 ± 0.31	10.41 ± 0.13	11.93 ± 0.24*	12.30 ± 0.13*
Liver weight/Body weight (g/kg)	24.8 ± 0.41	24.04 ± 0.30	31.64 ± 0.31*	32.38 ± 0.25*

Values are expressed as the mean ± SEM.

*P < 0.05 versus LS at the same time settings.

**Figure 6:**
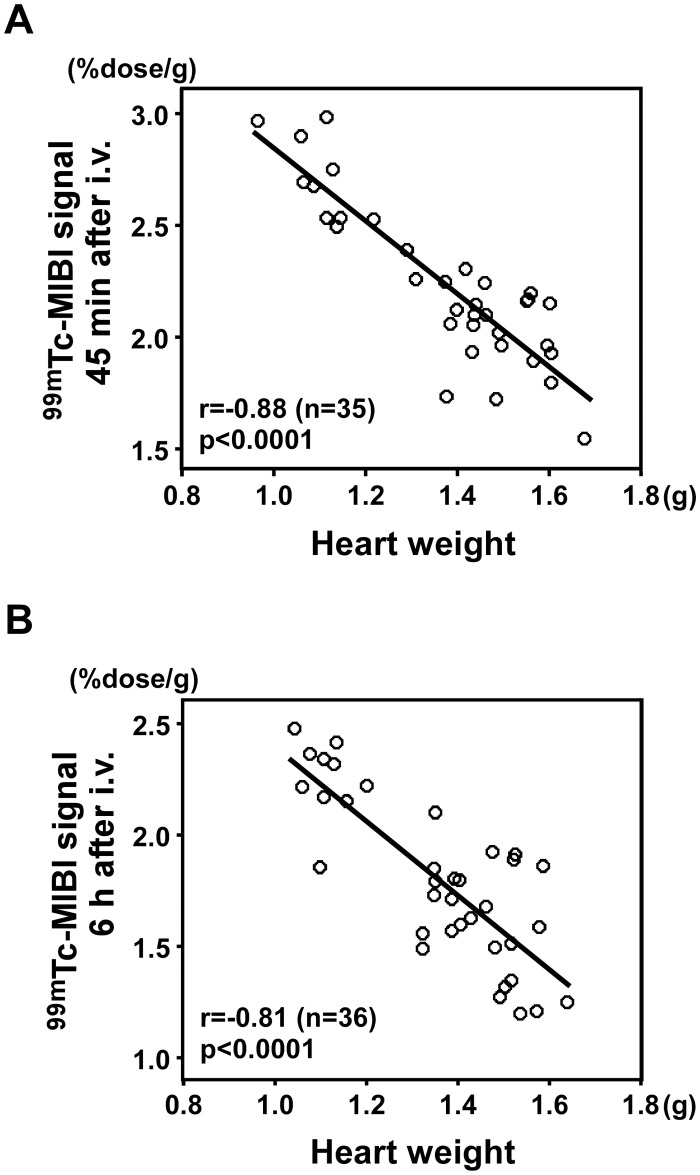
Correlation between ^99m^Tc-MIBI signals and heart weight. (A) **** ^99m^Tc-MIBI signal per gram of heart tissue 45 min after the injection was inversely correlated with heart weight (r = -0.88, n = 35, *P* < 0.0001). (B) ^99m^Tc-MIBI signal per gram of tissue 6 h after the injection inversely correlated with heart weight (r = -0.81, n = 36, *P* < 0.0001).

LS, rats fed a low-salt diet; HS, rats fed a high-salt diet;

**3–2. Correlation between ^99m^Tc-MIBI signals and heart weight.** The ^99m^Tc-MIBI signal per gram of tissue was inversely correlated with heart weight at both 45 min and 6 h after the injection (r = -0.88, n = 35, *P* < 0.0001, Fig. 6A; r = -0.81, n = 36, *P* < 0.0001, Fig. 6B, respectively).

**Figure 7:**
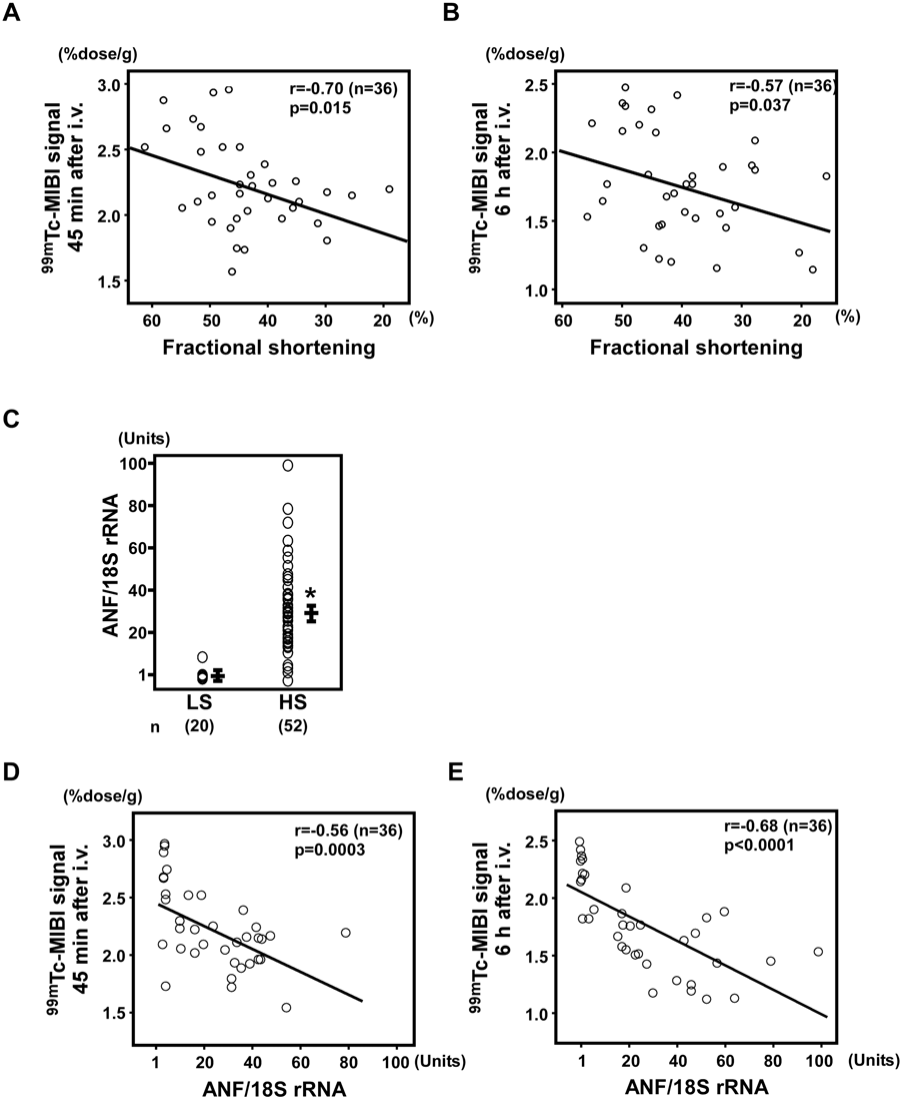
Correlation between ^99m^Tc-MIBI signals and cardiac function. (A, B) Correlation between fractional shortening on echocardiography and ^99m^Tc-MIBI signals 45 min after the injection (r = -0.70, n = 36, *P* = 0.015; A) or 6 h after the injection (r = -0.57, n = 36, *P* = 0.037; B). (C) Atrial natriuretic factor (*ANF)* gene expression was significantly increased in DS rats fed an HS diet (HS; n = 52) compared to DS rats fed an LS diet (LS; n = 20). *P < 0.05 versus LS. (D, E) Correlations between ANF gene expression and ^99m^Tc-MIBI signals 45 min (r = -0.56, n = 36, *P* = 0.0003; D) and 6 h (r = -0.68, n = 36, *P* < 0.00001; E) after the injection.

**3–3. Cardiac function and ^99m^Tc-MIBI signals.** Fractional shortening, as calculated by echocardiography, is the degree of shortening of the left ventricular diameter between end-diastole and end-systole and is used as an estimate of myocardial contractility. Using echocardiography, we calculated the fractional shortening of DS rats fed HS and LS diets. There was a correlation between fractional shortening and ^99m^Tc-MIBI signals (45 min after the injection: r = -0.70, n = 36, *P* = 0.015; 6 h after the injection: r = -0.57, n = 36, *P* = 0.037; Fig. 7A, 7B).

**3–4. Expression of *ANF* and ^99m^Tc-MIBI signals.** ANF is a hormone produced in the cardiac atrium in response to high blood volume [34], and expression of *ANF* mRNA has been shown to increases as a result of heart failure [35]. *ANF* gene expression was significantly increased in DS rats fed an HS diet (*P* < 0.001; Fig. 7C) and was correlated with ^99m^Tc-MIBI signals (45 min after the injection: r = -0.56, n = 36, *P* = 0.0003; 6 h after the injection: r = -0.68, n = 36, *P* < 0.00001; Fig. 7D, 7E).

**Figure 8:**
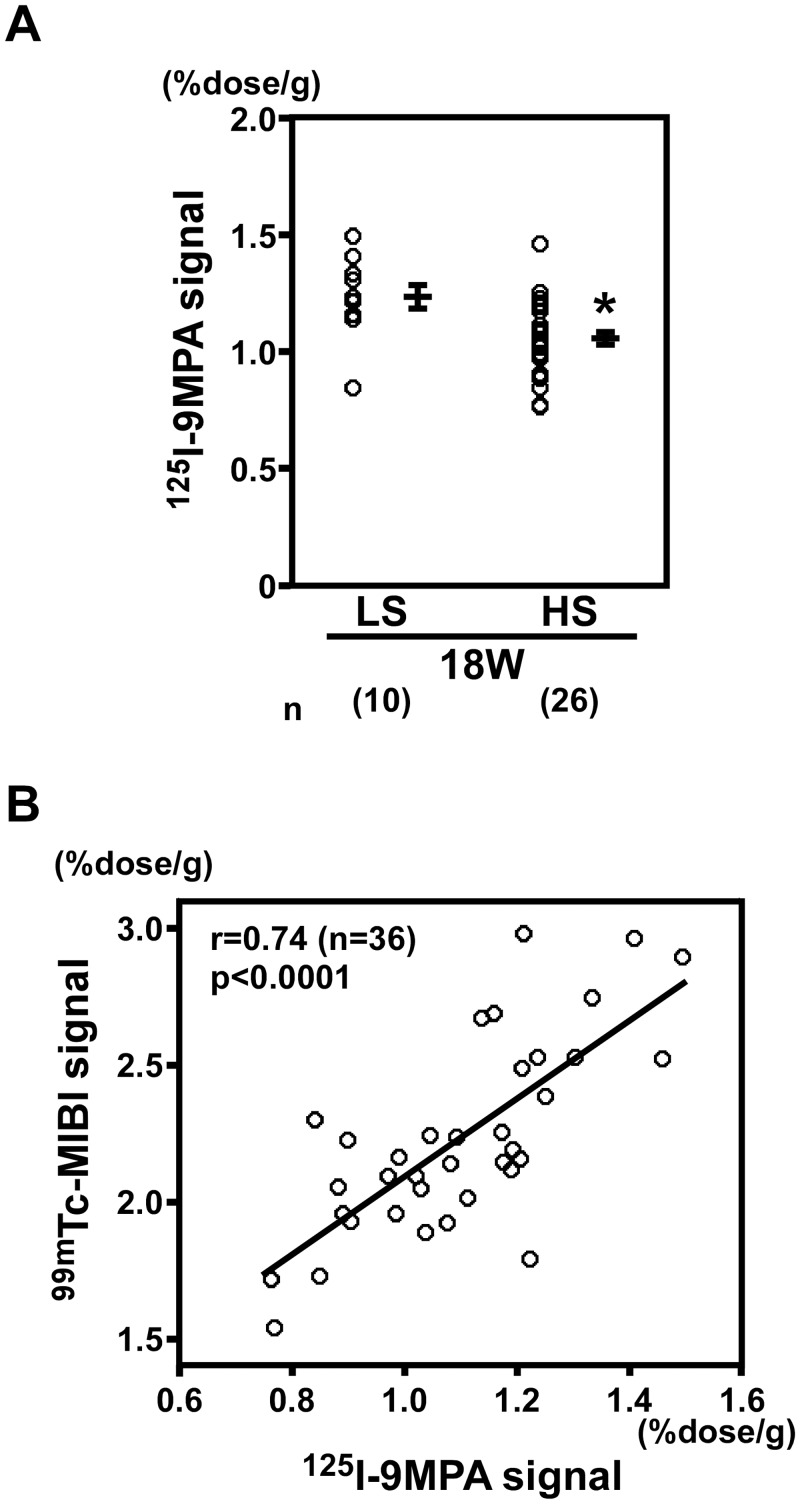
^125^I-9MPA signals and correlation with ^99m^Tc-MIBI signals. (A) In DS rats fed an HS diet (n = 26), the signals for ^125^I-9MPA, an indicator of the activity of mitochondrial β-oxidation, decreased compared with those in DS rats fed an LS diet (n = 10). *P < 0.05 versus LS. (B) ^125^I-9MPA signals positively correlated with ^99m^Tc-MIBI signals (r = 0.74, *P* < 0.0001, n = 36).

**3–5.^125^I-9MPA signals and correlation with ^99m^Tc-MIBI signals.** In DS rats fed an HS diet, the signals for ^125^I-9MPA, which indicate mitochondrial β-oxidation activity, decreased compared with those in DS rats fed an LS diet (*P* < 0.0001; Fig. 8A). ^125^I-9MPA signals were positively correlated with ^99m^Tc-MIBI signals (r = 0.74, n = 36, *P* < 0.0001; Fig. 8B), indicating that the decrease in ^99m^Tc-MIBI signals was associated with mitochondrial β-oxidation activity and the severity of heart failure.

**Figure 9:**
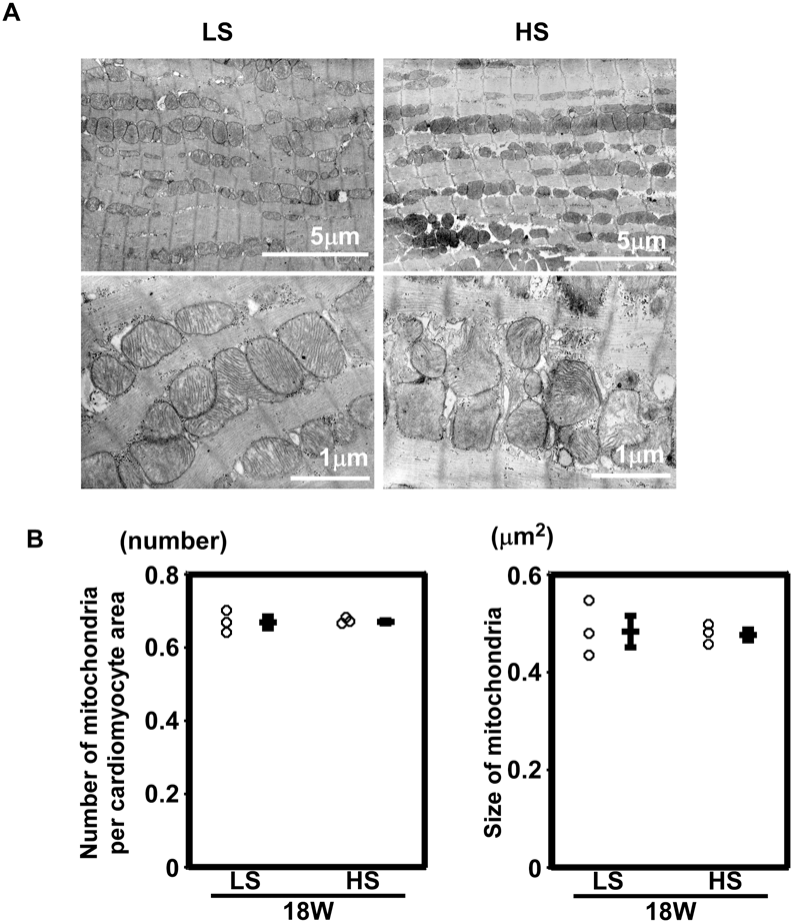
Mitochondrial density and morphology. (A) Low- and high- power electron micrographs from the left ventricles of rats fed an LS diet and an HS diet. (B) The density of mitochondria (number per 100 μm^2^) and the size of mitochondria (μm^2^) were not different between LS and HS group. n = 3 in each group.

**3–6. Mitochondrial density and morphology.** Electron microscopic analysis showed no apparent abnormalities between the hearts from rats fed an LS diet and those from rats fed an HS diet. Representative images are shown in Fig. 9A. The number and size of mitochondria did not differ significantly (n = 3 in each group, Fig. 9B). Neither the degeneration nor collapse of mitochondria was observed.

## Discussion

In the present study, we demonstrated that mitochondrial membrane potential could be analyzed using ^99m^Tc-MIBI signals with comparable sensitivity to TMRE in *ex vivo* perfused hearts. ^99m^Tc-MIBI signals decreased in the hearts of rats administered CCCP, and *in situ* ATP levels decreased accordingly. In a rat model of cardiac hypertrophy and failure, ^99m^Tc-MIBI signals per cardiac tissue weight inversely correlated with heart weight, cardiac function, and the expression of a marker of heart failure, and positively correlated with ^125^I-9MPA signals. The ^99m^Tc-MIBI signals in liver tissue were lower than those in heart tissue. A previous study showed that ^99m^Tc-MIBI signals in brown adipose tissue per tissue weight were inversely correlated with body weight in rats [36]. However, we verified that ^99m^Tc-MIBI signals could be used as a marker of mitochondrial membrane potential in *ex vivo* perfused hearts, and we investigated the ^99m^Tc-MIBI signals in an animal model, including simultaneous measurements in different organs.

Given that ^99m^Tc-MIBI has a high affinity for the negative charges associated with membrane potentials, the ^99m^Tc-MIBI signals measured in the present study may have been influenced by three possible factors: (1) the Nernstian equation across the plasma and mitochondrial membranes [2, 4]; (2) the volume of the mitochondrial matrix where the probe accumulates according to the Nerstian equation [3]; and (3) the amounts of mitochondrial inner membrane lipids if the probe binds to these lipids. When CCCP is acutely applied, the main effect is likely to be a depolarization of the mitochondrial membrane potential since phosphocreatine and ATP levels were significantly decreased. Our data in the SD rats indicated that measurement of **** ^99m^Tc-MIBI signals after acute administration of a mitochondrial uncoupler could be an effective tool for semiquantitative investigation of mitochondrial membrane potential in animals.

However, in rat models of chronic heart failure, it is possible that all three of the aforementioned factors are altered. Plasma membrane potential is defined by the concentration difference in sodium, potassium, and calcium across the membrane and the relative permeability of the membrane to each of these ions, which is regulated by ion channels. In heart failure, intracellular sodium is increased and, consequently, Ca^2+^ release decreases with the decreased efficiency of contraction [37]. Although we did not measure plasma membrane potential directly, it is possible that plasma membrane potential can be changed in DS rats with an HS diet. In terms of mitochondrial matrix volume, because the size, density, and morphology of mitochondria did not change between DS rats fed an LS diet and those fed an HS diet (Fig. 9), we speculated that the volume of the mitochondrial matrix did not change in this experiment.

The third possible factor affecting the ^99m^Tc-MIBI signals is mitochondrial inner membrane lipids, if the probe binds to these lipids. In DS rats with heart failure, the expressions levels of genes related to mitochondrial function were modified [21] [38]. In addition, mitochondrial membrane potential can be altered through the changes in the activity of components of the electron transport chain [39] and through the proton leaks by uncoupling proteins [21, 40] in animal models of heart failure. The ^99m^Tc-MIBI signals accumulate in proportion to succinate dehydrogenase (SDH) activity in the extracted heart [6]. The activity of succinate dehydrogenase and the expression of the *SDH* gene [39] are decreased during heart failure [41]. This was also supported by the observation that the ^99m^Tc-MIBI signal per tissue weight of the heart was positively correlated with signals for ^125^I-9MPA (a modified long chain (15 carbons, C-15) fatty acid). ^125^I-9MPA is well suited for studies on fatty acid metabolism and oxidation *in animals* [29–31]. Thus, our data showing that the ^99m^Tc-MIBI signal per heart tissue weight was reduced may indicate impaired mitochondrial function per tissue weight in the DS rats.

^99m^Tc-MIBI is used for the clinical diagnosis of coronary artery disease [42–44]. In a rat model of heart failure, coronary blood flow is the other possible confounding factor affecting ^9m^Tc-MIBI signals. We found that the ^99m^Tc-MIBI signal per heart tissue weight was inversely correlated with heart weight (Fig. 6A). One possible explanation is that coronary blood flow remains unchanged between hypertrophied hearts and normal hearts. Myocardial blood flow per left ventricle (LV) is similar in dogs with LVH and normal dogs [45]. Inversely, coronary blood flow (CBF) per myocardial mass decreases in hypertrophied hearts [46]. Consequently, CBF remains unchanged in patients with hypertensive LVH, but CBF per LV mass decreases significantly [47]. However, in the present study, we did not measure coronary blood flow of normal and failing hearts. The membrane potential-dependent net distribution of ^99m^Tc-MIBI signals is time-dependent. Cultured chick cardiomyocytes reach myocellular equilibrium at a t_1/2_ of 9.3 ± 1.5 min [48], and a blood clearance study showed that myocelluar equilibrium was reached at a t_1/2_ of 2–5 min in clinical use [49]. In our experiments, the blood concentration of ^99m^Tc-MIBI was supposed to be high (approximately 35 MBq/kg in bolus injection in rats) compared to that in clinical use (bolus injection of 370–550 MBq/person). If the concentration of ^99m^Tc-MIBI in the blood was maintained during an adequate period to reach myocellular equilibrium, coronary blood flow would not be important for determining the ^99m^Tc-MIBI signal; however CBF should be measured to clarify this point.

The ^99m^Tc-MIBI signals in rat livers were lower than those in rat hearts (Fig. 5B). The factors that may affect this result have been summarized in previous reports. Analyses of freeze-clamped rat liver tissue showed that the plasma membrane potential varies from 27 to 33 mV, as calculated from the measured metabolites [50], or measures around 56 mV, as calculated from the inorganic ions [50]; this is lower than the plasma membrane potential of the heart. In intact cells, values of mitochondrial membrane potential are between 143 and 161 mV in hepatocytes [51–53]. The mitochondrial membrane potential of hepatocytes does not seem to be lower than those of cardiomyocytes (117mV in embryonic cultured myocytes [4] and 100–140 mV in the perfused working heart [18]). In contrast, liver tissue contains fewer mitochondria per unit weight than heart tissue per unit weight [54, 55]. Electron microscopy also showed that mitochondria in liver tissue have poorer cristae in each mitochondrion, *i.e.*, have less membrane surface area, than those in the heart tissue, regardless of the species [56]. Our data demonstrate the reduced summation of the mitochondrial matrix volume, mitochondrial membrane potentials, and other factors, such as properties of mitochondrial inner membrane lipids, per liver tissue weight.

A number of *in vitro, ex vivo,* and *in situ* methods for the assessment of mitochondrial function have been used, each with strengths and weaknesses. These methods have produced inconsistent analyses of mitochondrial function, which may be due to the range of experimental conditions employed and the different functions observed. There is an inconsistency between the measured capacity for oxidant generation using isolated mitochondria and the measurements of total ROS markers *in an animal model* of altered cardiac metabolism [57]. The observed changes in mitochondrial function (respiratory function or ATP synthesis) in permeabilized cells do not always correlate with the morphological changes of mitochondria [8, 13, 58, 59]. These inconsistencies are likely the result of making measurements of specific functions or morphologies in purified mitochondria and cells, which lack cellular and extracellular factors that interact with the mitochondria in working organs and *in situ.* Considering the importance of mitochondria in various diseases [7, 9], the assessment of mitochondria in animals is thought to be important. Measurements of ^99m^Tc-MIBI signals in animals could serve as an assessment of mitochondrial function, and mitochondrial membrane potential if confounding factors that affect the ^99m^Tc-MIBI signals in the tissue are carefully evaluated and adjusted.

In conclusion, measurement of **** ^99m^Tc-MIBI signals can be an effective tool for semi-quantitative investigation of cardiac mitochondrial membrane potentials in SD rats in which a chemical is used to decrease the mitochondrial membrane potential. In our study, the ^99m^Tc-MIBI signal per tissue weight was reduced in the Dahl rat model of heart failure, although the precise mechanism is still unknown.

### Limitations of this study

The ^99m^Tc-MIBI signal was not measured in isolated mitochondria in the present study. We did not measure the membrane potential of plasma and mitochondria, nor the properties of inner membrane lipids, or the function of isolated mitochondria in the Dahl rat model, and we did not measure the CBF or the kinetics of ^99m^Tc-MIBI in the present study. Therefore, we could not determine whether the decreased ^99m^Tc-MIBI signal per tissue weight of the heart showed a decline in the mitochondrial membrane potential in the Dahl rat model.
